# Tegaserod Stimulates 5-HT_4_ Serotonin Receptors in the Isolated Human Atrium

**DOI:** 10.3390/ijms252011133

**Published:** 2024-10-17

**Authors:** Christin Hesse, Joachim Neumann, Valerie Compan, Evgeni Ponimaskin, Franziska E. Müller, Britt Hofmann, Ulrich Gergs

**Affiliations:** 1 Institute for Pharmacology and Toxicology, Medical Faculty, Martin Luther University Halle-Wittenberg, 06097 Halle, Germany; christin.hesse@uk-halle.de (C.H.); ulrich.gergs@medizin.uni-halle.de (U.G.); 2 Brains’ Laboratory Lsco, Department of Sciences, Nîmes University, 30021 Nîmes, France; valerie.compan@unimes.fr; 3 Cellular Neurophysiology, Center of Physiology, Hannover Medical School, 30625 Hannover, Germany; ponimaskin.evgeni@mh-hannover.de (E.P.); mueller.franziska@mh-hannover.de (F.E.M.); 4 Department of Cardiac Surgery, Mid-German Heart Center, University Hospital Halle, 06097 Halle, Germany; britt.hofmann@uk-halle.de

**Keywords:** tegaserod, human atrium, mouse atrium, cardiac serotonin receptors

## Abstract

Tegaserod (1-{[(5-methoxy-1H-indol-3-yl)methyliden]amino}-3-pentylguanidine) is a potent agonist at human recombinant 5-HT_4_ serotonin receptors. Consequently, tegaserod is utilized in the treatment of bowel diseases. The objective of this study was to test the hypothesis that tegaserod stimulates human cardiac atrial 5-HT_4_-receptors via cyclic adenosine monophosphate (cAMP)-dependent pathways. Tegaserod exerted positive inotropic effects (PIEs) and positive chronotropic effects (PCEs) in isolated left and right atrial preparations, respectively, from mice with cardiac-specific overexpression of the human 5-HT_4_ serotonin receptor (5-HT_4_-TG) in a concentration- and time-dependent manner. However, no effect was observed in the hearts of littermates of wild-type mice (WT). Western blot analysis revealed that the expression of 5-HT_4_ receptors was significantly higher in 5-HT_4_-TG mice compared to WT mice. The specificity of the signal for the 5-HT_4_ receptor was confirmed by the absence of the signal in the hearts of 5-HT_4_ receptor knockout mice. Furthermore, tegaserod increased the force of contraction (at concentrations as low as 10 nM), reduced the time of tension relaxation, and increased the rate of tension development in isolated electrically stimulated (at a rate of 60 beats per minute) human right atrial preparations (HAPs, obtained during open-heart surgery) when administered alone. The potency and efficacy of tegaserod to raise the force of contraction were enhanced in the presence of cilostamide, a phosphodiesterase III inhibitor. The positive inotropic effect of tegaserod in HAPs was found to be attenuated by the 5-HT_4_ serotonin receptor antagonist GR 125487 (0.1 µM). The efficacy of tegaserod (10 µM) in raising the force of contraction in HAPs was less pronounced than that of serotonin (10 µM) or isoprenaline (1 µM). Tegaserod shifted the concentration–response curve of the force of contraction to serotonin to the right in HAPs, indicating that it is a partial agonist at 5-HT_4_ serotonin receptors in this model. We propose that the mechanism of action of tegaserod in HAPs involves cAMP-dependent phosphorylation of cardiac regulatory proteins.

## 1. Introduction

**Figure 1:**
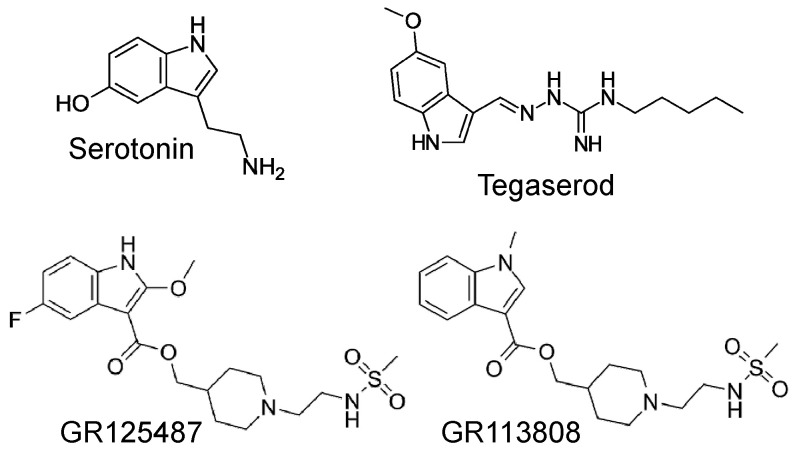
Structural formulae of serotonin, tegaserod, GR 125487, and GR 113808, all used in this study.

Tegaserod is a chemical compound that belongs to the indole carbazimidamide class (Figure 1) [1] and has been demonstrated to stimulate gastric 5-HT_4_ receptors [2]. Tegaserod is approximately four times more potent than serotonin (5-HT) in stimulating 5-HT_4_ receptors in the gastrointestinal tract [3]. Stimulation of the 5-HT_4_-receptors in the gastrointestinal tract is suitable for treating symptoms of irritable bowel diseases like constipation [2]. Accordingly, tegaserod was developed for the treatment of irritable bowel disease and its beneficial effects are likely the result of direct stimulatory effects on 5-HT_4_ receptors in the gut smooth muscle and/or indirect effects via local acetylcholine release from motor neurons in the gut [2]. In contrast to other drugs, such as the chemically distinct benzamides cisapride and metoclopramide, tegaserod exhibits the advantage of lacking interactions with D_2_ dopamine receptors, 5-HT_3_ serotonin receptors, or potassium channels [3,4]. Nevertheless, in electrophysiological experiments, 3 µM tegaserod (a concentration exceeding the therapeutic range) was observed to inhibit potassium currents (hERG-currents in transfected CHO cells) [5].

The adverse cardiovascular effects, including unstable angina, myocardial infarction, and stroke, resulted in the temporary withdrawal of tegaserod from the market in numerous countries [6]. However, subsequent clinical studies have disproven the hypothesis that tegaserod increases the risk of cardiac complications. Consequently, tegaserod was reintroduced to the market, at least in the United States [7]. Some have proposed that the cardiac side effects of tegaserod are not mediated by cardiac 5-HT_4_ receptors, suggesting that tegaserod may be inactive at the cardiac 5-HT_4_ receptors in humans. It is possible that tegaserod does not signal in the heart because the affinity of agonists at 5-HT_4_ receptors is known to be organ dependent. This means that the affinity may be different in the human atrium than in the human gut due to the presence of organ-specific splice variants of 5-HT_4_ receptors. This has been discussed in several reviews [3,8,9]. Indeed, whether tegaserod was a full or partial agonist in the gut was species dependent [10]. In vitro, tegaserod bound to human 5-HT_4_, 5-HT_2B_, 5-HT_2A_, 5-HT_7_, 5-HT_3A_, 5-HT_5A_, 5-HT_6_, and 5-HT_1_ receptors with decreasing affinity [11]. Therefore, tegaserod exhibits a binding affinity to the majority of 5-HT receptors. Nevertheless, the highest affinity for tegaserod is observed for 5-HT_4_ and 5-HT_2B_ receptors, with a Ki-value of 8.2 for both receptors. However, the positive inotropic effects of 5-HT in the human heart are solely mediated via the 5-HT_4_ receptors [12,13,14,15]. We therefore considered it important to investigate the effects of tegaserod on the human atrium in more detail.

Studies have demonstrated the presence and functionality of 5-HT_4_ receptors in the human atrium [13,14] and diseased human ventricle [16,17,18]. In animals, only pig hearts and monkey hearts express 5-HT_4_ receptors that increase the force of contraction and the beating rate of the heart [9,19]. We have generated and characterized transgenic mice with overexpression of functional human 5-HT_4_ receptors in the heart [20]. The 5-HT_4_-TG model has been used to detect the cardiac effects of 5-HT_4_ receptor agonists, including prucalopride, cisapride, metoclopramide, and bufotenin [21,22,23].

In summary, the following hypotheses were tested: Firstly, tegaserod stimulates the force of contraction of isolated left atrial preparations from 5-HT_4_-TG and stimulates the beating rate in spontaneously beating right atrial preparations from 5-HT_4_-TG. Secondly, tegaserod stimulates the force of contraction in isolated human atrial preparations via 5-HT_4_ receptors.

## 2. Results

**Figure 2:**
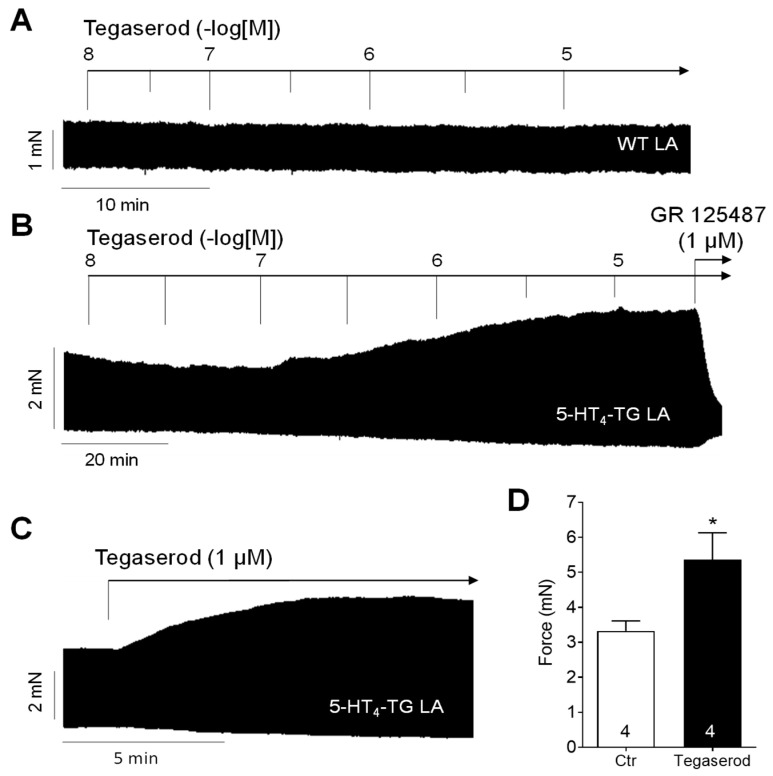
(**A**) Original recording of cumulatively applied tegaserod in an isolated, electrically stimulated left atrial preparation (LA) from a wild-type (WT) mouse. (**B**) Original recording of a mouse left atrial preparation as in A, but from 5-HT_4_-TG. Tegaserod induced a time- and concentration-dependent positive inotropic effect only in 5-HT_4_-TG that is antagonized by the 5-HT_4_ receptor antagonist GR 125487. (**C**) Original recording of a left atrial preparation of a 5-HT_4_-TG mouse. Tegaserod was given in a single dosage. (**D**) Summarized data (mean ± SEM) of force of contraction in mouse left and right atrial preparations with application of 1 µM tegaserod (Tega). * *p* = 0.125 (Wilcoxon matched-pairs signed rank test) versus control (Ctr; pre-drug value). The numbers in bars indicate the number of experiments. Ordinates are in millinewton (mN).

Tegaserod exerted a concentration- and time-dependent positive inotropic effect in left atrial preparations from 5-HT_4_-TG (Figure 2B). In contrast, tegaserod was ineffective in raising the force of contraction in left atrial preparations from WT (Figure 2A). Furthermore, the application of GR 125487, a 5-HT_4_ receptor antagonist, completely reversed the positive inotropic effect of tegaserod in 5-HT_4_-TG (Figure 2B). The results are consistent with our previous findings. 5-HT is unable to elevate force of contraction in atrial preparations from WT (see, for example, [20]). Figure 2C shows the typical original recording for the effect of a single application of 1 µM tegaserod in 5-HT_4_-TG and in Figure 2D, several of these experiments are summarized. Force of contraction was increased by 59% from 3.33 ± 0.28 mN to 5.36 ± 0.77 mN (n = 4), but this effect was not statistically significant (non-parametric Wilcoxon test) due to the low number of experiments. For comparison, the force of contraction of the spontaneously beating right atrial preparations was also evaluated, but because of the negative force–frequency relationship in mouse atria, no positive inotropic effect of tegaserod was noted.

**Figure 3:**
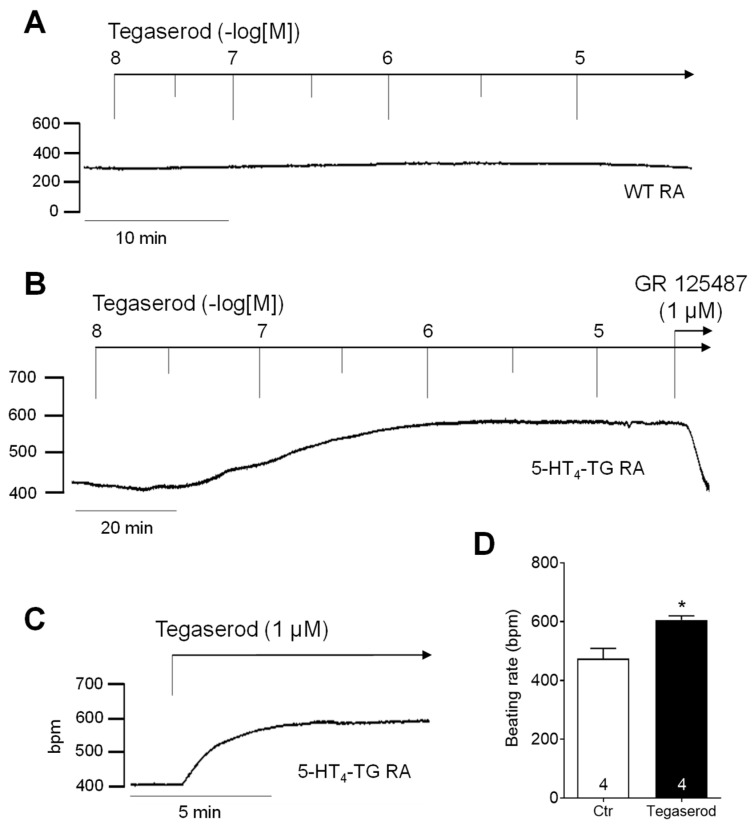
(**A**) Original recording of cumulatively applied tegaserod in an isolated, spontaneously beating right atrial preparation (RA) from a wild-type (WT) mouse. (**B**) Original recording of a mouse right atrial preparation as in A, but from 5-HT_4_-TG. Tegaserod induced a time- and concentration-dependent positive chronotropic effect only in 5-HT_4_-TG that is antagonized by GR 125487. (**C**) Original recording of a right atrial preparation of a 5-HT_4_-TG mouse. Tegaserod was given in a single dosage. (**D**) Summarized data (mean ± SEM) of the beating rate in mouse right atrial preparations with application of 1 µM tegaserod (Tega). * *p* = 0.125 (Wilcoxon matched-pairs signed rank test) versus control (Ctr; pre-drug value). The numbers in bars indicate the number of experiments. Ordinates are in beats per minute (bpm).

If tegaserod behaves similarly to 5-HT in this model system, it would be expected to augment the beating rate in the right atrium of 5-HT_4_-TG. Indeed, a time- and concentration-dependent positive chronotropic effect of tegaserod was observed, as shown in Figure 3, which presents the original recordings of the beating rate in right atrial preparations from WT (Figure 3A) and 5-HT_4_-TG (Figure 3B). The effect of a single application of tegaserod (1 µM) in 5-HT_4_-TG is shown in Figure 3C as an original recording and summarized for several experiments in Figure 3D. The beating rate was increased from 474 ± 35 bpm to 606 ± 14 bpm (n = 4), but correspondingly to the left atrial force, this effect was not statistically significant (non-parametric Wilcoxon test) due to the limited number of experiments.

**Figure 4:**
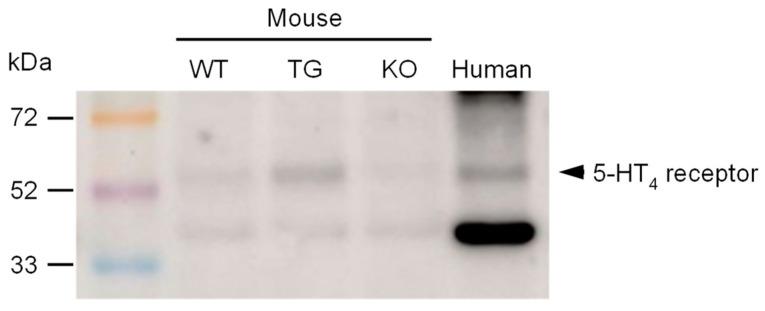
A representative Western blot for the 5-HT_4_ receptor antibody is shown herewith. The molecular weight markers (rainbow marker) are indicated in kilodaltons (kDa). The following samples were loaded: ventricular homogenates of a wild-type mouse (WT), a 5-HT_4_-TG mouse (TG), a 5-HT_4_ knock-out mouse (KO), and a homogenate of a human right atrial sample (human).

Furthermore, we investigated the expression of 5-HT_4_ receptors in transgenic animals in comparison to wild-type controls. To ensure the validity of the results, a negative control was used in the form of hearts from 5-HT_4_ receptor knockout mice. As shown in Figure 4, the 5-HT_4_ receptor is overexpressed in transgenic mice. Additionally, the 5-HT_4_ receptor was identified in a human atrial sample (Figure 4 and border-bottom:solid thin;border-left:solid thin;border-right:solid thin;).

**Figure 5:**
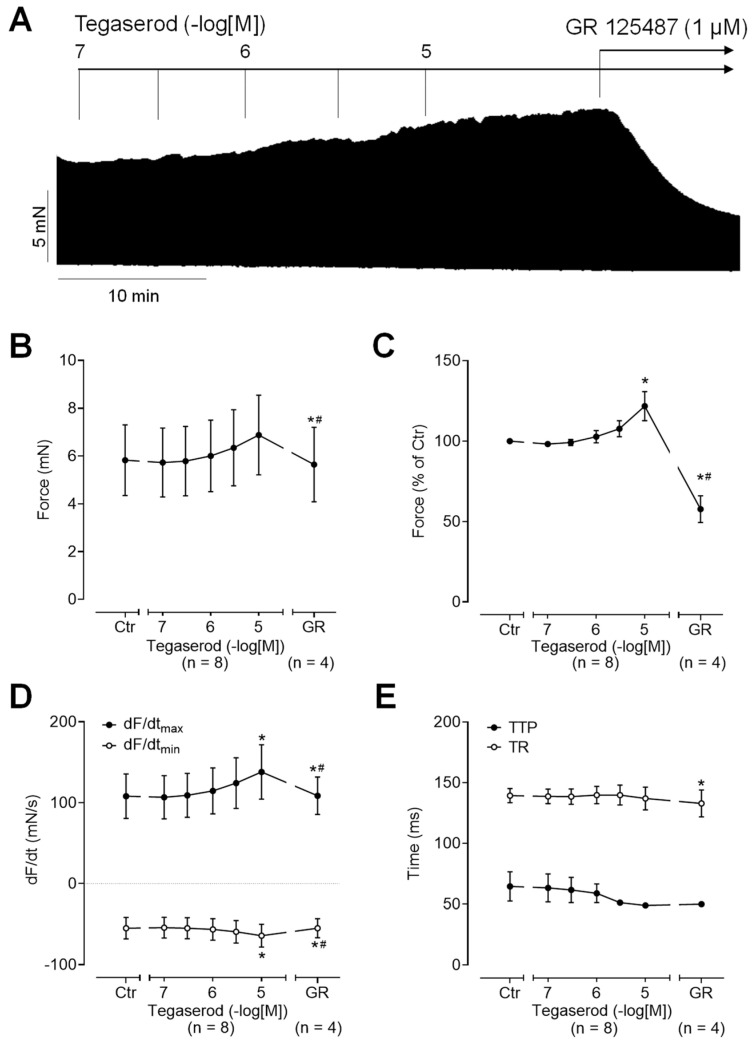
(**A**) Original recording of the concentration- and time-dependent positive inotropic effect of tegaserod in a human right atrial preparation in millinewtons (mN). The horizontal bar indicates the time axis in minutes (min). (**B**–**E**) The summarized effects (means ± SEM) of cumulatively applied tegaserod on the force of contraction in millinewtons (mN) (**B**) or in percentage of the pre-drug value (Ctr) (**C**) and on the rate of tension development (dF/dtmax) and rate of relaxation (dF/dtmin) in millinewtons per second (mN/s) (**D**) and on the time to peak tension (TTP) and time to relaxation (TR) in milliseconds (ms) (**E**) are presented. The effects of tegaserod (n = 8) were antagonized by 1 µM of the 5-HT_4_ receptor antagonist GR 125487 (GR, n = 4). * *p* < 0.05 vs. Ctr (pre-drug value); # *p* < 0.05 vs. 10 µM tegaserod (mixed-effects analysis with Bonferroni’s multiple comparisons test). The numbers in parentheses indicate the number of experiments.

**Figure 6:**
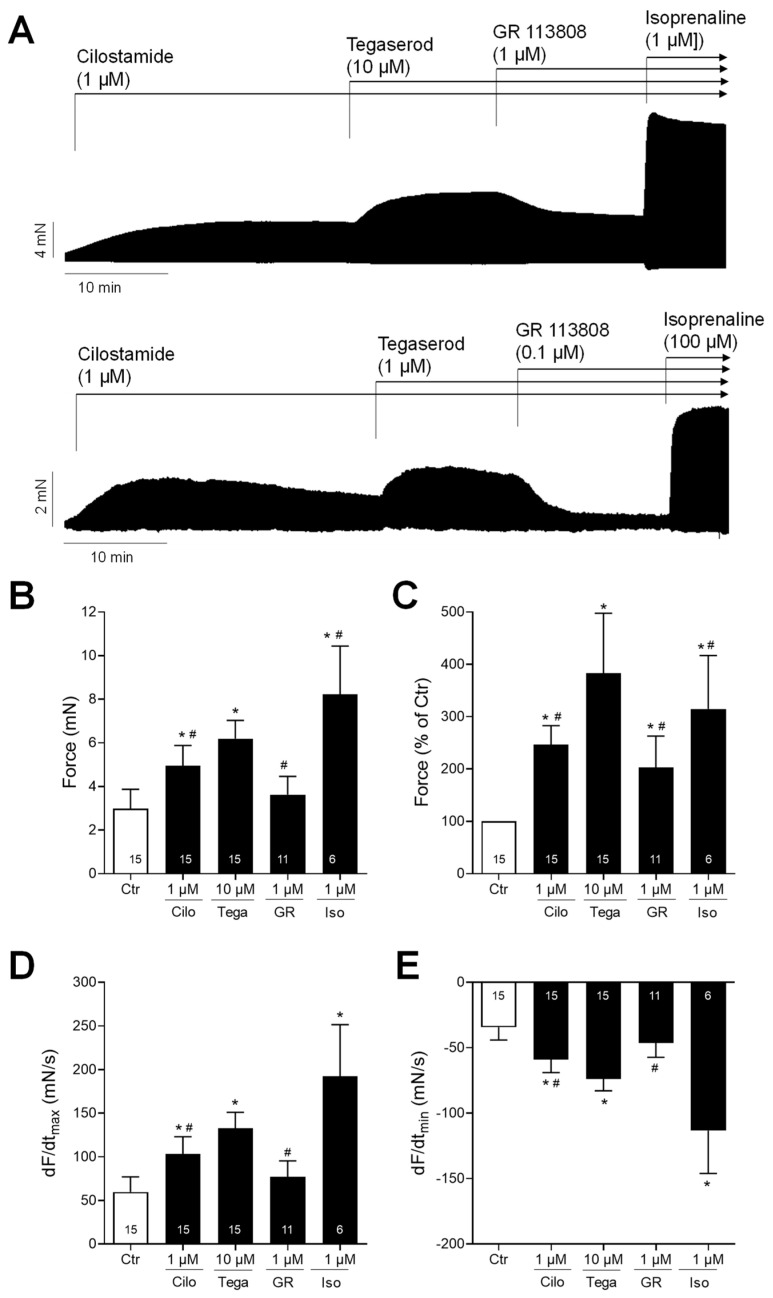
(**A**) Original recordings of the concentration- and time-dependent positive inotropic effect of tegaserod in millinewtons (mN) after cilostamide in human right atrial preparations. The horizontal bars indicate the time axis in minutes (min). First, 1 µM cilostamide was added followed by tegaserod; then, the 5-HT_4_ receptor antagonist GR 113808 (GR) was added. Finally, at the end of the experiment, isoprenaline (Iso) was added to the organ bath to test for efficiencies. (**B**–**E**) The summarized effects (means ± SEM) of 10 µM tegaserod (Tega) in the presence of cilostamide (Cilo; 1 µM) on the force of contraction in millinewtons (mN) (**B**) or in percentage of the pre-drug value (Ctr) (**C**) and on the rate of tension development (dF/dtmax) (**D**) and rate of relaxation (dF/dtmin) (**E**) in millinewtons per second (mN/s) are presented. Additionally, the effects of 1 µM GR and 1 µM Iso are shown. * *p* < 0.05 vs. Ctr (pre-drug value); # *p* < 0.05 vs. 10 µM tegaserod (mixed-effects analysis with Bonferroni’s multiple comparisons test). The numbers in bars indicate the number of experiments.

Next, we wanted to test the effects of tegaserod in the human heart. To this end, we mounted human right atrial preparations in the organ bath, stimulated them electrically, and obtained concentration–response curves for tegaserod. As demonstrated by the original recording in Figure 5A, the application of tegaserod resulted in a time- and concentration-dependent increase in the force of contraction. This increase was attenuated by the subsequent application of GR 125487. The inotropic effects of tegaserod (0.1–10 µM) are summarized (n = 8) in Figure 5B–E. In half of the right atrial preparations, the 5-HT_4_ receptor antagonist GR 125487 (1 µM) was administered following the administration of tegaserod. The data for force are presented in millinewtons (mN) in Figure 5B and as a percentage of the pre-drug value (Ctr) in Figure 5C. As illustrated in Figure 5C, the normalized data indicate a 25% increase in force of contraction when tegaserod is applied alone. Tegaserod increased both the rate of tension development and the rate of tension relaxation (Figure 5D). The administration of tegaserod did not result in a reduction in the time required to reach peak tension or the time required for relaxation (Figure 5E). Furthermore, tegaserod was more effective and potent to increase the force of contraction in HAPs when they were first stimulated by cilostamide and then tegaserod was added (Figure 6A). Following the testing of varying drug concentrations (Figure 6A), the administration of 1 µM cilostamide followed by 10 µM tegaserod, 1 µM GR 113808, and finally 1 µM isoprenaline produced the most pronounced effects in the majority of atrial preparations. The data from 15 preparations could be summarized for the effects of tegaserod. Unfortunately, not all the preparations were sufficiently stable to undergo evaluation following the application of GR 113808 (n = 11) or isoprenaline (n = 6). The data for the effects of tegaserod on force of contraction in millinewtons (mN), in percentage of pre-drug value, and on the rate of tension development and rate of relaxation are presented in Figure 6B–E. In conclusion, tegaserod increased the force parameters (Figure 6B–E) in the additional presence of cilostamide. These effects were antagonized by GR 113808, a 5-HT_4_ receptor antagonist (Figure 6B–E). A comparison of the results presented in Figure 5 and Figure 6 reveals the impact of pre-stimulation by phosphodiesterase inhibition, thereby underscoring the significance of cAMP-dependent processes in the signaling of tegaserod.

**Figure 7:**
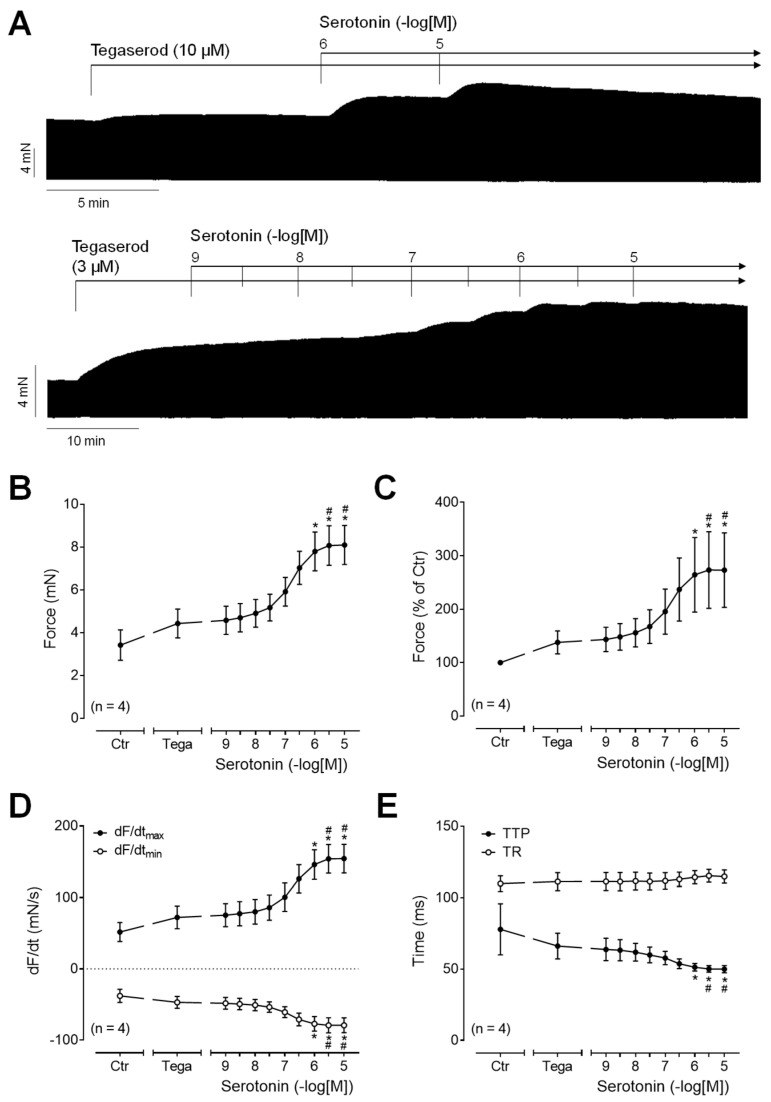
(**A**) Original recordings of the concentration- and time-dependent positive inotropic effect of a single concentration of tegaserod before cumulatively applied serotonin in millinewtons (mN) in human right atrial preparations. Horizontal bars indicate time axis in minutes (min). (**B**–**E**) The summarized effects (means ± SEM) of cumulatively applied serotonin in presence of 10 µM tegaserod (Tega) on the force of contraction in millinewtons (mN) (**B**) or in percentage of the pre-drug value (Ctr) (**C**) and on the rate of tension development (dF/dtmax) and rate of relaxation (dF/dtmin) in millinewtons per second (mN/s) (**D**) and on the time to peak tension (TTP) and time to relaxation (TR) in milliseconds (ms) (**E**) are presented. * *p* < 0.05 vs. Ctr (pre-drug value); # *p* < 0.05 vs. 10 µM tegaserod (ANOVA: non-parametric Friedman test with Dunn’s multiple comparisons test). The numbers in parentheses indicate the number of experiments.

To test whether tegaserod functions as a full 5-HT_4_ receptor agonist in human atrial preparations, serotonin was applied either after or before the administration of tegaserod. Following the application of tegaserod, an additional application of serotonin resulted in a further time- and concentration-dependent increase in the force of contraction (Figure 7A: original recording). The effects, measured in four independent preparations, are quantified in Figure 7B–E. It is apparent that serotonin in absolute values (Figure 7B) or in percentage of pre-drug value (Figure 7C) increased the force of contraction after pre-treatment with tegaserod (10 µM). Similarly, after tegaserod, serotonin increased the rate of tension development and the rate of relaxation (Figure 7D). The time parameters remained partly unaffected (Figure 7E). These findings suggest that tegaserod exerts a partial agonistic effect on the human right atrium.

**Figure 8:**
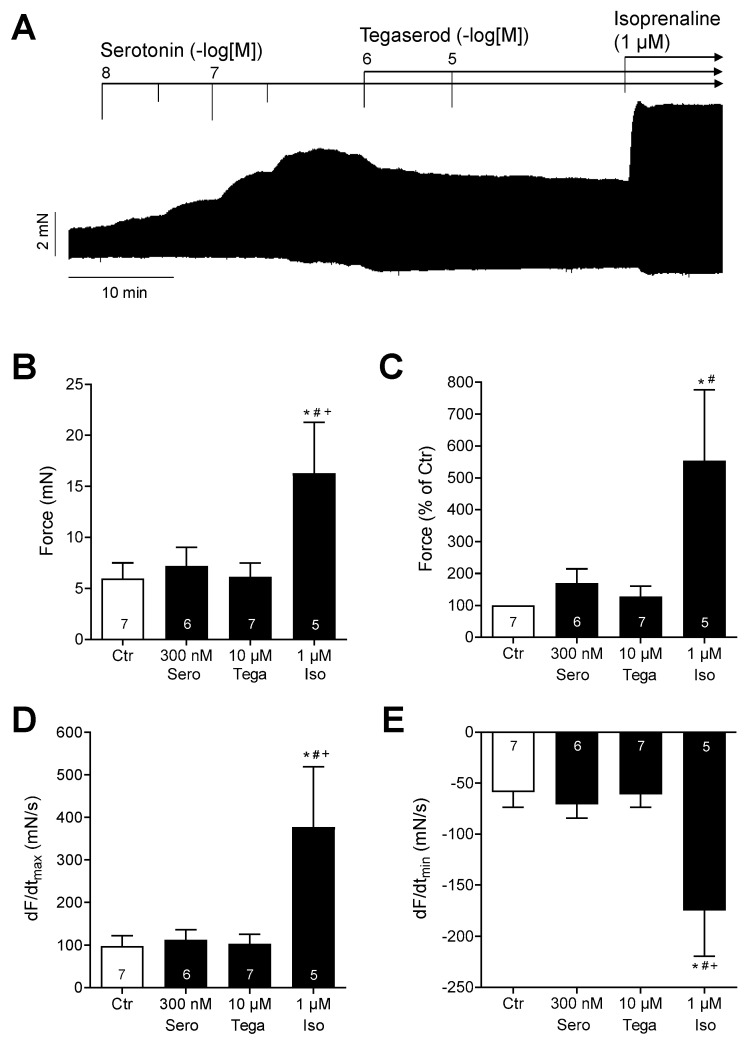
(**A**) Original recording of the concentration- and time-dependent positive inotropic effect of tegaserod (1 µM and 10 µM) after cumulatively applied serotonin (up to 300 nM) in millinewtons (mN) in a human right atrial preparation. Horizontal bar indicates time axis in minutes (min). At the end of the experiment, 1 µM isoprenaline (Iso) was added to the organ bath to test for efficiencies. (**B**–**E**) The summarized effects (means ± SEM) of 10 µM tegaserod (Tega) in the presence of serotonin (Sero; 300 nM) on the force of contraction in millinewtons (mN) (**B**) or in percentage of the pre-drug value (Ctr) (**C**) and on the rate of tension development (dF/dtmax) (**D**) and rate of relaxation (dF/dtmin) (**E**) in millinewtons per second (mN/s) are presented. Additionally, the effect of finally applied Iso (1 µM) is shown. * *p* < 0.05 vs. Ctr (pre-drug value); # *p* < 0.05 vs. 10 µM tegaserod; + *p* < 0.05 vs. 300 nM serotonin (mixed-effects analysis with Bonferroni’s multiple comparisons test). The numbers in bars indicate the number of experiments.

However, in the presence of maximally effective serotonin, additionally administered tegaserod reduced the contraction force in HAPs (Figure 8A: original recording). The data from several experiments (n = 5–7: not all preparations could be evaluated) are summarized in Figure 8B,C for force, and Figure 8D,E for the rate of tension development and rate of relaxation. It should be noted that isoprenaline was more effective than serotonin in raising the force of contraction (Figure 8). Although the effects of serotonin and tegaserod were not statistically significant in this evaluated experimental group, it can be postulated that tegaserod may act as an inverse agonist in the presence of serotonin in the human right atrium. However, this remains speculative at present.

## 3. Discussion

The main new findings are as follows. The use of transgenic mice demonstrated that tegaserod increases the force of contraction only when 5-HT_4_ receptors are overexpressed in the heart. Tegaserod acts as a full agonist in the atria of these mice. The efficacy and potency of tegaserod to increase the force of contraction are potentiated by a phosphodiesterase inhibitor. Tegaserod shortens the rate of relaxation and increases the rate of tension development and rate of relaxation in isolated human atrial preparations. These data collectively suggest that tegaserod exerts its effects in the human heart by increasing the content of cAMP.

### 3.1. Cardiac Effects of Tegaserod in Different Models

It was reported that tegaserod exerted positive chronotropic and inotropic effects in isolated atrial preparations of pigs [24]. Tegaserod was as effective as serotonin to raise the force of contraction in isolated right atrial preparations from pigs. However, tegaserod was less effective than serotonin to raise the force of contraction in isolated left atrial preparations from pigs, suggesting the presence of regional differences [24].

In isolated, paced human atrial preparations, tegaserod increased the force of contraction [25] and these contractile effects of tegaserod were blocked by GR 113808, a 5-HT_4_ receptor antagonist [25]. Tegaserod demonstrated reduced efficacy compared to serotonin in human atrial preparations [25]. Furthermore, tegaserod shifted the concentration dependent positive inotropic effect of serotonin in human right atrial preparations to higher concentrations of serotonin [25]. This suggests a partial agonistic action of tegaserod in the human atrium.

In isolated human atrial preparations, 100 nM of tegaserod augmented the I_f_-current, a current that plays a role in the initiation of the heartbeat [26]. Therefore, it can be postulated that tegaserod may exert direct chronotropic effects on the human heart. Tegaserod at concentrations of up to 10 µM was ineffective in inducing contraction of the isolated left anterior descending arteries from the human heart [27]. This was interpreted as beneficial because 5-HT via other receptors constricts human coronaries, an unwanted side effect that is thus unlikely to occur with tegaserod therapy. It is noteworthy that in certain studies, tegaserod demonstrated a higher affinity for human 5-HT_2B_ or 5-HT_2A_ serotonin receptors than for the human 5-HT_4_ receptors [27]. Additionally, higher concentrations of tegaserod were observed to bind to the human serotonin transporter (SERT) and to the rat noradrenaline or dopamine transporter [28], as well as to the hERG [27]. These data suggest that tegaserod may enhance the inotropic and chronotropic effects of noradrenaline or serotonin in the human heart. This is because, like cocaine, tegaserod impairs the uptake of these neurotransmitters, thereby increasing their sarcolemmal concentration. Furthermore, if tegaserod were to inhibit hERG in the human heart, this would result in a prolongation of the monophasic action potential, consequently prolonging the duration of the total contraction time in human atrial preparations. These effects have not been studied before.

### 3.2. Mechanism of Tegaserod

It is proposed that tegaserod increased the force of contraction as an agonist at cardiac human 5-HT_4_ receptors, based on the observation that tegaserod only increased contractility in the atrium of 5-HT_4_-TG mice, and not in wild-type mice. A comparison of the concentration–response curves of tegaserod and serotonin [20] in mouse atrial preparations indicates that tegaserod at 5-HT_4_ receptors in the left and right atria acts as a full agonist in the transgenic mouse. This is not the case in the human atrium. In the human atrium, tegaserod is a partial agonist, exhibiting reduced efficacy compared to serotonin (in accordance with the findings of Chai et al., 2012 [25]). Tegaserod has been demonstrated to antagonize the positive inotropic effect of serotonin in HAPs [25]. This discrepancy may be attributed to the higher expression of 5-HT_4_ receptors in transgenic mice compared to the human atria. An alternative explanation is that the discrepancy may be attributed to differential signal transduction in human hearts in comparison to 5-HT_4_-TG. In accordance with the preceding report, tegaserod also acted as an agonist at 5-HT_4_ receptors in the isolated human atrium [25]. This interpretation is based on the observation that the positive inotropic effect of tegaserod is attenuated by the antagonists GR 125487 and GR 113808. Therefore, it can be concluded that the in vivo effects of tegaserod on the force of contraction in the human heart are mediated by 5-HT_4_ receptors. However, the efficacy of tegaserod in the human heart was less pronounced than that of serotonin or isoprenaline. It is known that isoprenaline is more effective than serotonin in raising the force of contraction in the human heart. It should be noted that this is not a general phenomenon. In 5-HT_4_-TG, serotonin and isoprenaline are equally effective at raising the force of contraction (left atrium) or the beating rate (right atrium), likely due to the higher expression of the transgenic 5-HT_4_ receptors compared to human receptors (see Western blot). In the human atrium, serotonin is approximately 50% as effective as a stimulation of β-adrenoceptors [13,29].

### 3.3. Role of Phosphorylation of Regulatory Proteins

**Figure 9:**
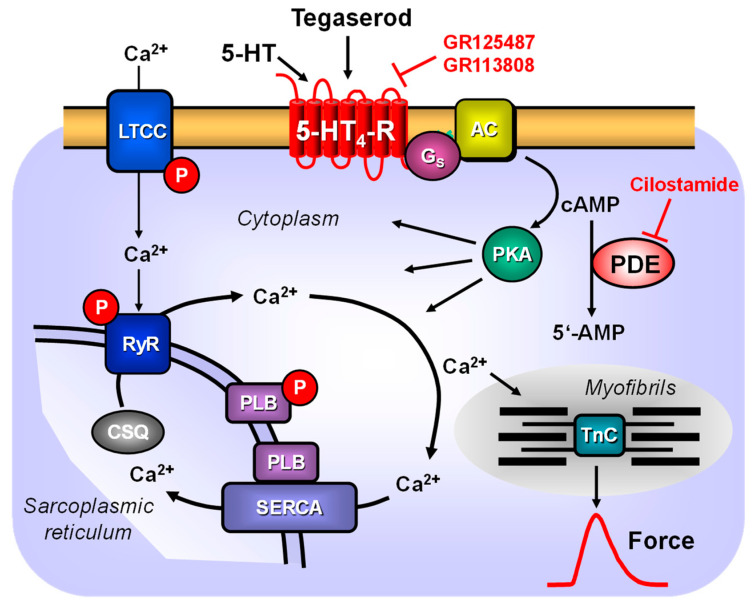
Scheme: the mechanisms of action of serotonin and tegaserod in cardiomyocytes. A heptahelical 5-HT_4_ serotonin receptor (5-HT_4_-R) is illustrated within the sarcolemma. The agonist serotonin (5-HT) activates the 5-HT_4_ serotonin receptor. Consequently, the stimulatory G-protein (Gs) augments the ability of adenylyl cyclases (AC) to generate cAMP. This cAMP can activate cAMP-dependent protein kinases (PKA). Subsequently, PKA phosphorylates and activates target proteins, including the L-type calcium channel (LTCC) in the sarcolemma and the ryanodine receptor (RyR) in the sarcoplasmic reticulum (SR). The phosphorylation of phospholamban has been demonstrated to increase the activity of the SR-calcium ATPase (SERCA). In the human heart, mainly the phosphodiesterase isoform III (PDE 3) converts cAMP to the inactive 5′-AMP. The PDE 3 is inhibited by cilostamide. Tegaserod and serotonin may activate human cardiac 5-HT_4_ serotonin receptors.

With regard to the signal transduction system utilized by tegaserod, it is probable that the 5-HT_4_ receptor system is utilized in a manner consistent with the illustration depicted in Figure 9. Moreover, the effects of tegaserod were potentiated by an inhibitor of phosphodiesterase III (cilostamide), which is the predominant phosphodiesterase in the human heart. This suggests, in addition, that the inhibition of SERT by tegaserod observed in cell culture studies [28] may not be translated into a functional response. One would have expected that, as with cocaine, tegaserod would have potentiated the effect of serotonin; however, this was not the case. The general assumption is that stimulation of the 5-HT_4_ receptor results in an increase in the phosphorylation state of proteins that are substrates for the cAMP-dependent protein kinases (Figure 9). Our group was the first to demonstrate that serotonin, acting via 5-HT_4_ receptors, can enhance the phosphorylation state of phospholamban in the isolated human atrium, consequently increasing the activity of SERCA [29]. These phosphorylations provide a potential explanation for the observed increase in relaxation rate in human atrial preparations following the administration of tegaserod [30,31]. This perspective is supported by findings with thapsigargin, which inhibits the activity of SERCA. This inhibitory action of thapsigargin could indicate that tegaserod acts via the involvement of SERCA [30]. Furthermore, the observation that nifedipine, an antagonist at the L-type Ca^2+^ channel (LTCC) [32], can attenuate the effects of tegaserod is consistent with the role of protein phosphorylation. The phosphorylation of LTCC by cAMP-dependent protein kinase can facilitate the passage of Ca^2+^ through the LTCC, which in turn triggers the release of Ca^2+^ from the sarcoplasmic reticulum via the ryanodine receptor (Figure 9). Our findings align with the observation that tegaserod increased the Ca^2+^ current through LTCC in isolated human atrial cardiomyocytes [33].

### 3.4. Practical Aspects of Using Tegaserod

It is hypothesized that any tachycardia and potentially atrial fibrillation that may occur in patients following treatment with tegaserod could be effectively managed with tropisetron, an approved pharmaceutical agent. Nevertheless, this hypothesis requires testing in a clinical trial. Peak therapeutic plasma levels of tegaserod in patients have been reported to reach 6.3 µg/L (approximately 20 nM) following oral administration of 12 mg of the drug [34]. In a prior study, the administration of 100 mg of tegaserod resulted in a plasma concentration of approximately 120 nM and a bioavailability of approximately 10% [1]. Therefore, as inotropic effects were observed in human atrial preparations at 10 nM, direct effects may be a relevant factor. Furthermore, the evidence suggests that tegaserod is subject to partial degradation in humans [34]. Two-thirds of orally administered tegaserod are excreted unchanged in the feces, while one-third is renally eliminated. Tegaserod is hydrolyzed to an aldehyde, which is then oxidized by CYP2D6 [34] and to a lesser extent by CYP3A isoenzymes [35] to 5-methoxy-indole-carboxylic acid, which is then glucuronidated and eliminated renally [3,10]. As renal function declines with age, the elimination half-life of tegaserod has been observed to increase in the elderly [34]. Additionally, the elderly are more susceptible to developing atrial fibrillation. Furthermore, numerous drugs have been shown to inhibit the metabolism of tegaserod, potentially leading to increased plasma concentrations and elevated cardiac side effects of tegaserod.

### 3.5. Limitations

Due to the study design, the results are limited to the effects of tegaserod on isolated atrial preparations. It is therefore unclear to what extent the results can be extrapolated to the entire heart in the living organism. In particular, for humans, it could be argued that the effects on the sinus node of the human have not been tested directly. In order to do so, access to the human pacemaker would be required. However, such studies were beyond the scope of this initial study. Furthermore, the opportunity to study contractility and phosphorylation in human ventricle tissue was not possible due to a lack of access to that tissue.

## 4. Materials and Methods

### 4.1. Mouse Models

#### 4.1.1. 5-HT_4_-TG Mice

The mice used in this study were generated in-house and exhibited cardiac myocyte-selective overexpression of the human 5-HT_4_ serotonin receptor (5-HT_4_-TG) and littermate wild-type (WT) controls. The details on generation and characterization of the 5-HT_4_-TG mice was described previously [20]. All mice were handled in accordance with the local regulatory guidelines.

#### 4.1.2. 5-HT_4_R-KO Mice

Hearts from 5-HT_4_R-knockout mice of the strain B6-Htr4tm1Comp were used as control tissue along with wild-type littermates in Western blot experiments. The mice were housed and cared for in accordance with the directive 2010/63/EU and local regulations. All procedures performed on animals were conducted in accordance with the guidelines of the European Commission (European Communities Council Directive 2010/63/EU) and the United Kingdom Home Office (Scientific Procedures) Act (1986). The following mouse strains were used in this study: C57BL6/J Htr4 wild-type (WT) and knockout (KO) mice were obtained and described in detail by Compan et al., 2004 [36].

### 4.2. Contractile Studies on Mouse Preparations

This study utilized five male and one female mice with an average age of 156 ± 39 days. In brief, the right or left atrial preparations from the mice were isolated and mounted in organ baths as previously described [20]. For all organ bath experiments, a physiological salt solution at 37 °C was used containing 119.8 mM NaCl, 5.4 mM KCl, 1.8 mM CaCl_2_, 1.05 mM MgCl_2_, 0.42 mM Na_H_PO_4_, 22.6 mM NaHCO_3_, 0.05 mM Na_2_EDTA, 0.28 mM ascorbic acid, and 5.05 mM glucose, which was continuously gassed with 95% O_2_ and 5% CO_2_ to maintain a pH of 7.4 [20]. The magnitude of the force of contraction was determined in electrically paced, isolated left atrial preparations. The electrical stimulation was applied for a duration of five milliseconds with a rectangular impulse of direct current. The voltage was set at 10% above the threshold required to initiate the contraction, and the stimulation frequency was set at one beat per second (1 Hz). The preparations were stretched to their maximum basal force and then allowed to stabilize for 30 min before any drug application was initiated. The study of any chronotropic effects was conducted using spontaneously beating right atrial preparations in mice. The signals were measured using a PowerLab system together with the software LabChart Pro 8.0 (AD Instruments, Bella Vista, Australia).

### 4.3. Contractile Studies on Human Preparations

The contractile studies on human right atrial preparations were performed using the identical setup and buffer as used in the aforementioned mouse left atrial studies. The samples were obtained from nine male patients and four female patients, aged 64–82 years. The pharmacological treatment included metoprolol, furosemide, apixaban and acetyl salicylic acid. The methods used for atrial contraction studies in human samples have been previously published and were not altered in this study, e.g., [23,29].

### 4.4. Western Blotting

The homogenization of the samples, protein measurements, electrophoresis, primary and secondary antibody incubation and quantification were performed in accordance with previously established protocols [20,29]. In contrast to the previously published protocols, precast gradient gels (Novex^TM^ 4–20% “Tris-Glycine Plus Midi Protein Gels”, Invitrogen, by Thermo Fisher Scientific, Waltham, MA, USA) were used and the bound antibodies were visualized by chemiluminescence (Immobilon^TM^, Millipore, by Merck, Darmstadt, Germany) and an imaging system (Amersham ImageQuant 800, Cytiva, Freiburg im Breisgau, Germany). The primary antibody utilized was Anti-5-HT_4_ receptor (HTR4) antibody #ASR-036 (Alomone Labs, Jerusalem, Israel), diluted 1:1000. A total of 10 µg of protein was loaded per lane.

### 4.5. Data Analysis

Data shown are means ± standard error of the mean (SEM). Statistical significance was estimated using the analysis of variance (ANOVA or mixed-effects analysis in the case of missing values) followed by Bonferroni’s multiple comparisons test (human data). For limited numbers of experiments, the non-parametric Friedman test followed by Dunn’s multiple comparisons test (human data) or the non-parametric Wilcoxon matched-pairs signed rank test (mouse data) were used. All statistical analyses and the preparation of graphs were conducted using the software Prism 9.0 (GraphPad Software, San Diego, CA, USA). A *p*-value < 0.05 was considered to be significant.

### 4.6. Drugs and Materials

The drugs isoprenaline-hydrochloride, tegaserod, and serotonin were purchased from Sigma-Aldrich (Taufkirchen, Germany). The drugs GR 125487, GR 113808, and cilostamide were purchased from Tocris/Bio-Techne (Wiesbaden, Germany). A 10 mM stock solution of tegaserod was prepared in dimethyl sulfoxide (DMSO) and kept at –25 °C. All other chemicals were of the highest purity grade commercially available. Deionized water was used throughout the experiments. Stock solutions were prepared fresh daily.

## 5. Conclusions

In conclusion, the hypotheses presented in the introduction can now be addressed as follows: tegaserod was found to increase the force of contraction and beating rate in 5-HT_4_-TG. Furthermore, tegaserod elevated the force of contraction in the human atrium via 5-HT_4_ receptors through a cAMP-dependent pathway.

## Data Availability

The raw data supporting the conclusions of this article will be made available by the authors on request.

## Supplementary Materials

The following supporting information can be downloaded at: https://www.mdpi.com/article/10.3390/ijms252011133/s1.
