# Supplementary figures and images for: Tegaserod Stimulates 5-HT4 Serotonin Receptors in the Isolated Human Atrium
Source: Int J Mol Sci. 2024 Oct 17;25(20):11133. doi: 10.3390/ijms252011133 (PMC11508481; doi:10.3390/ijms252011133)

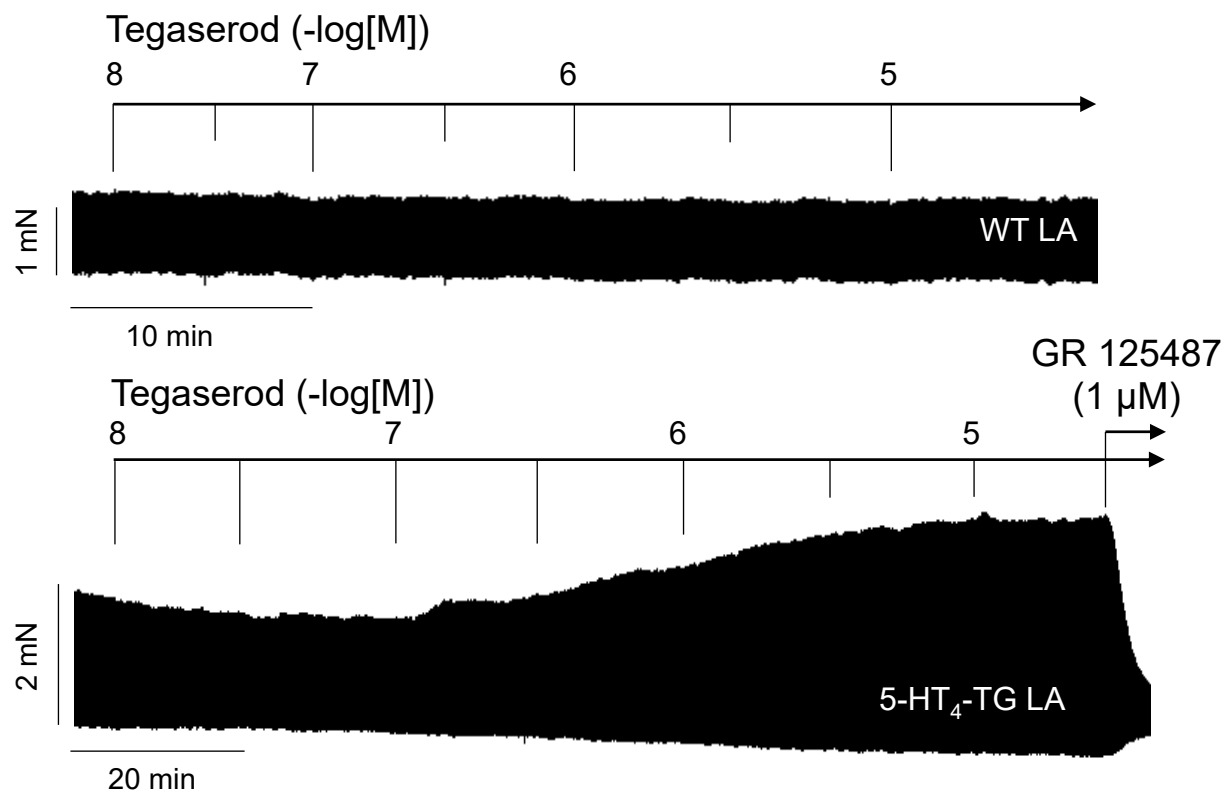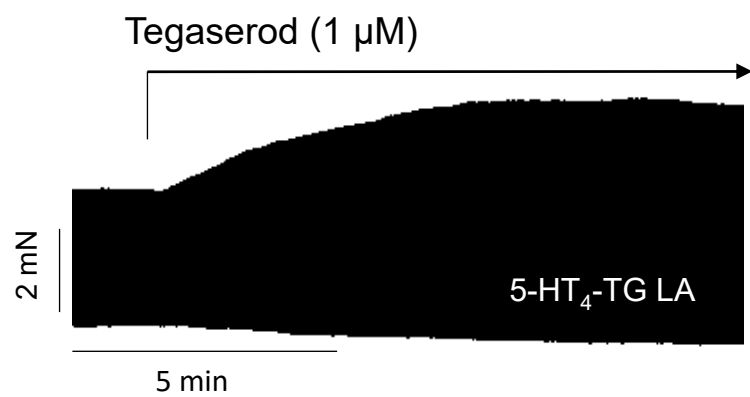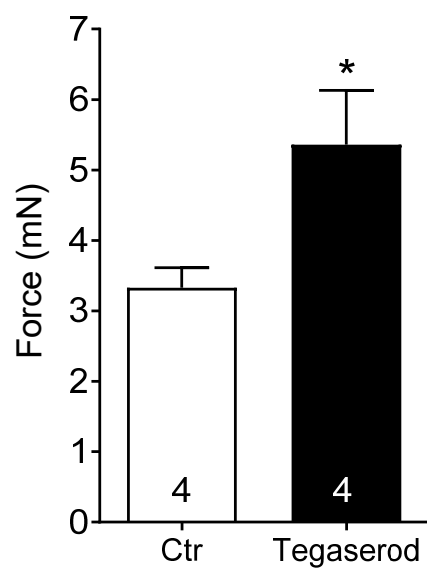

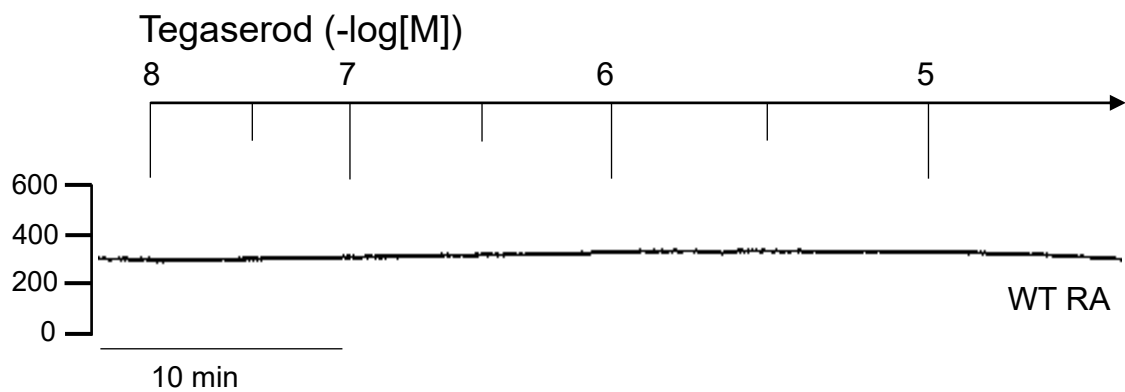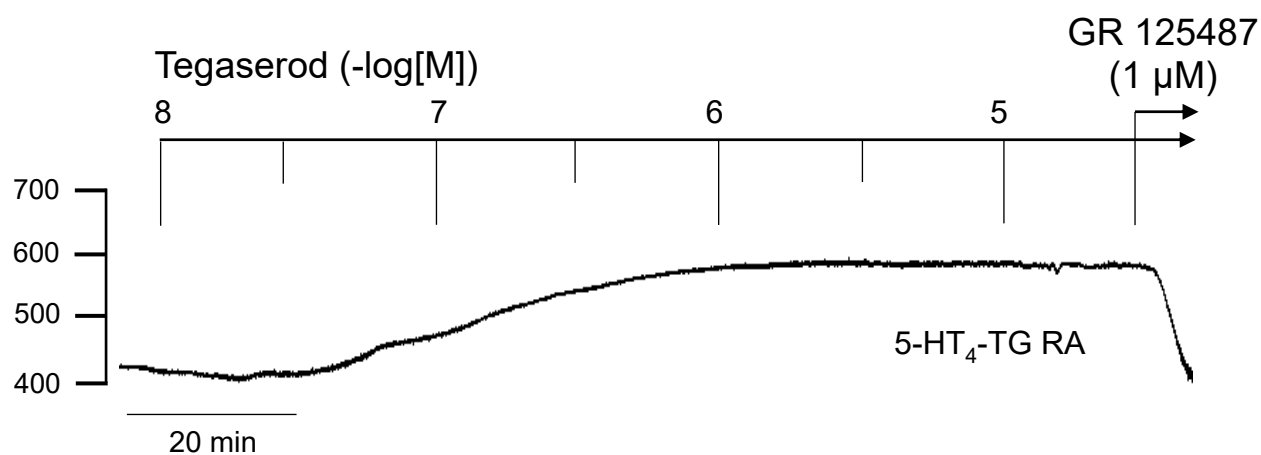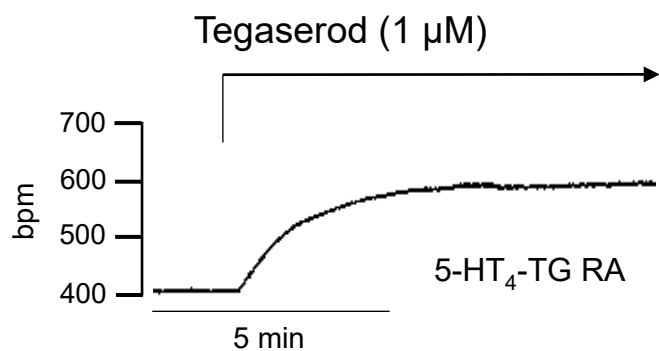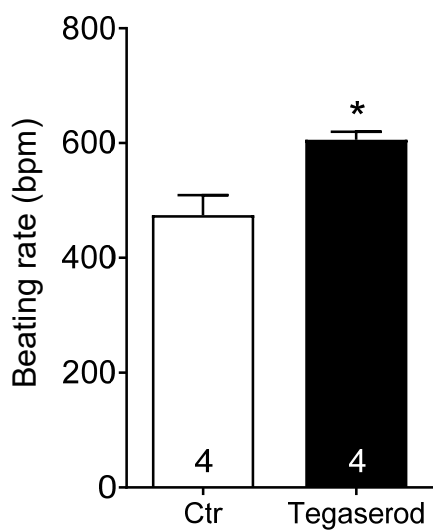

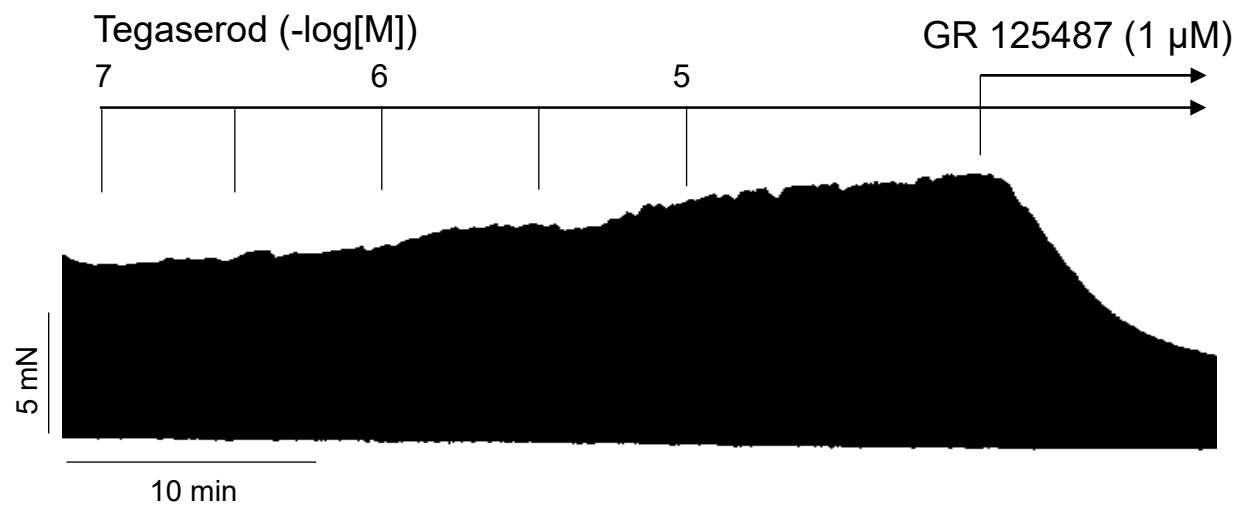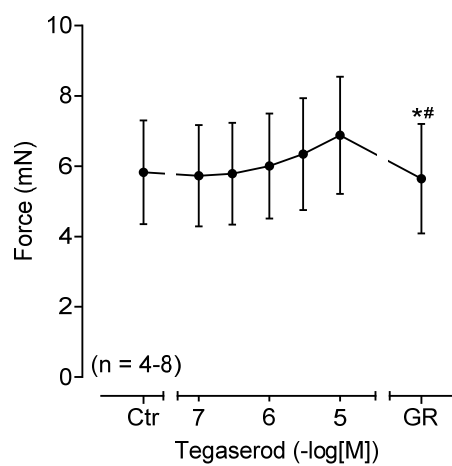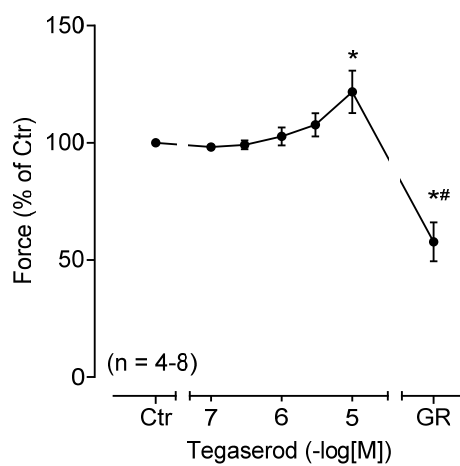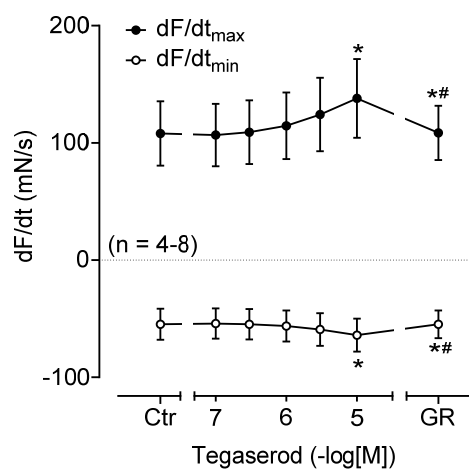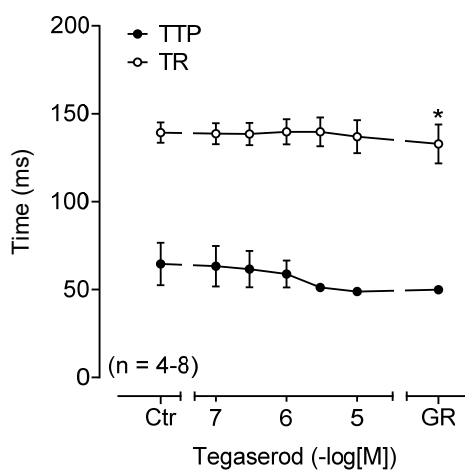

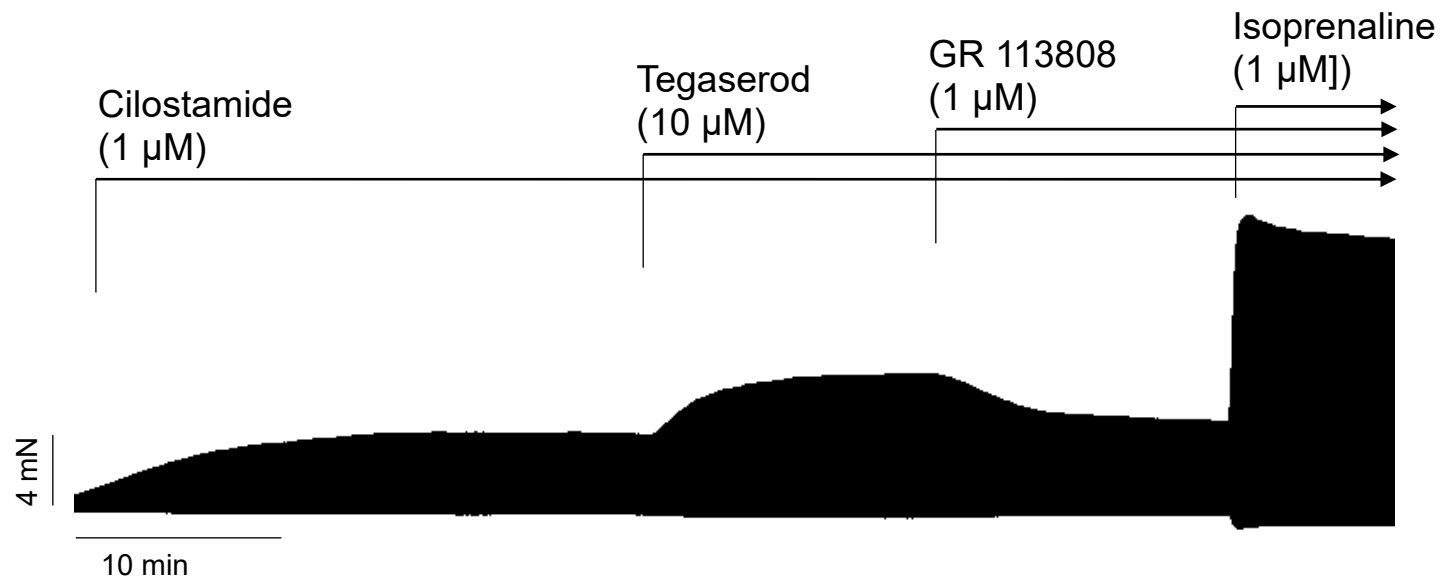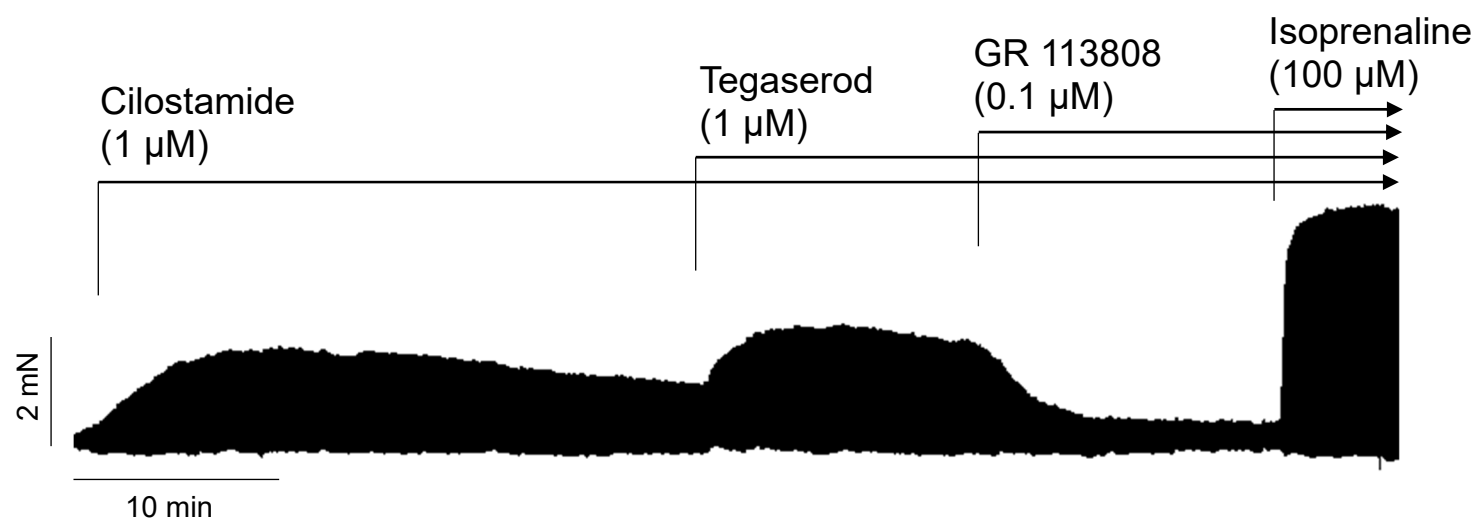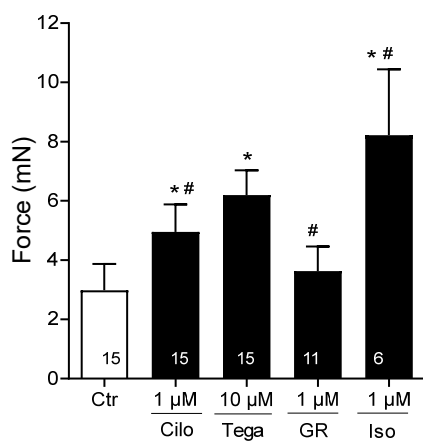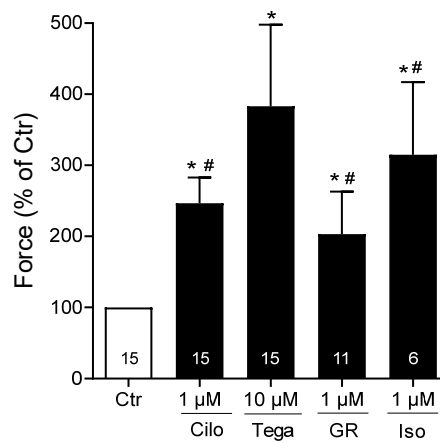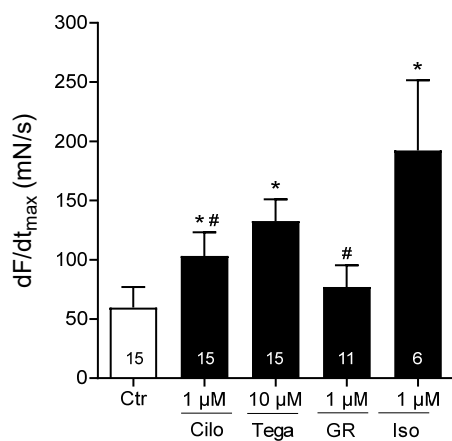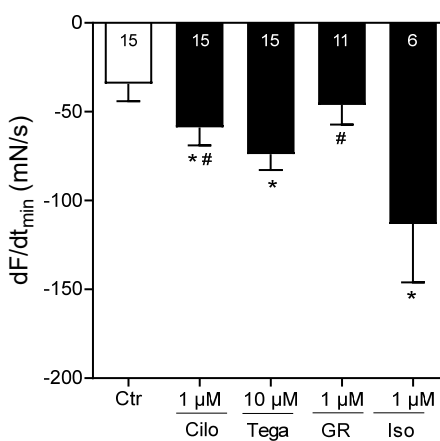

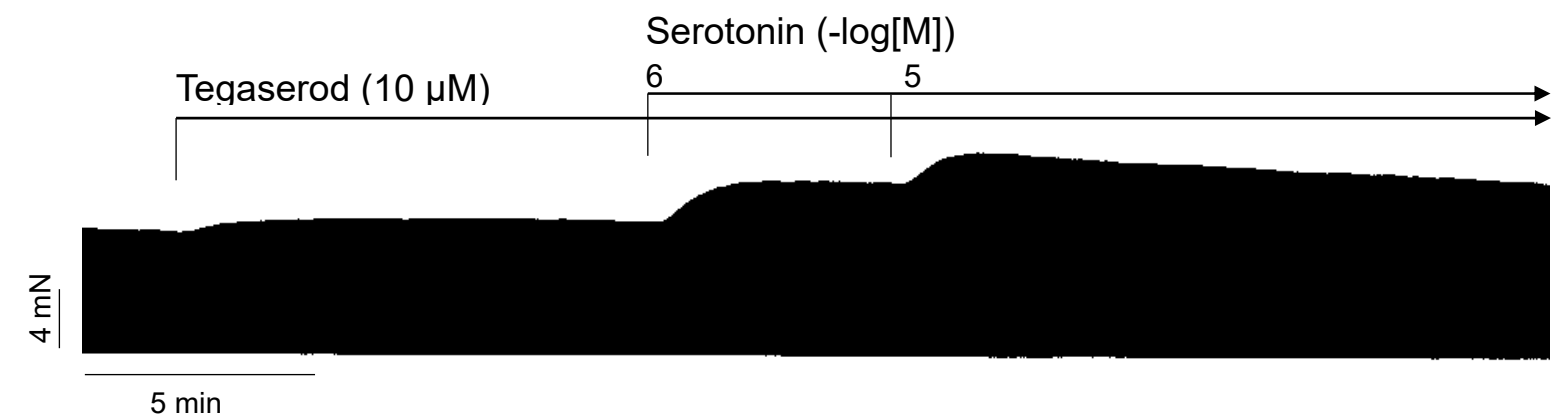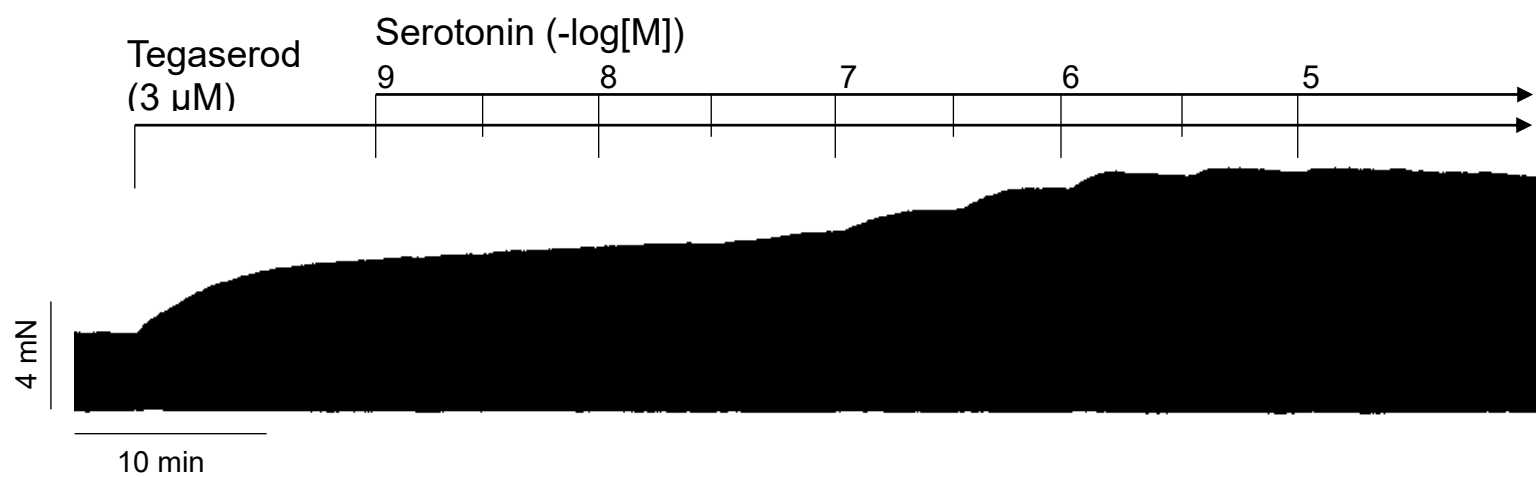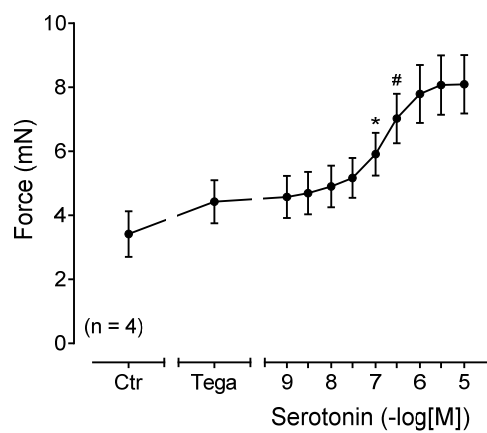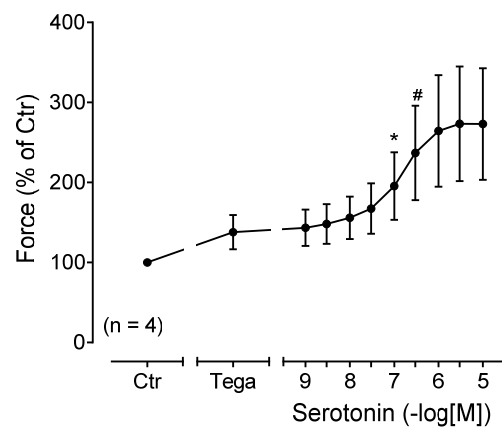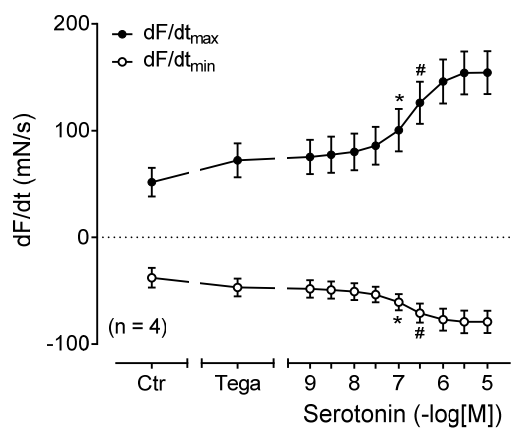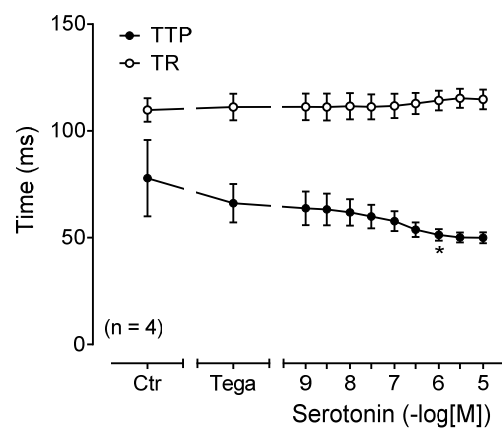

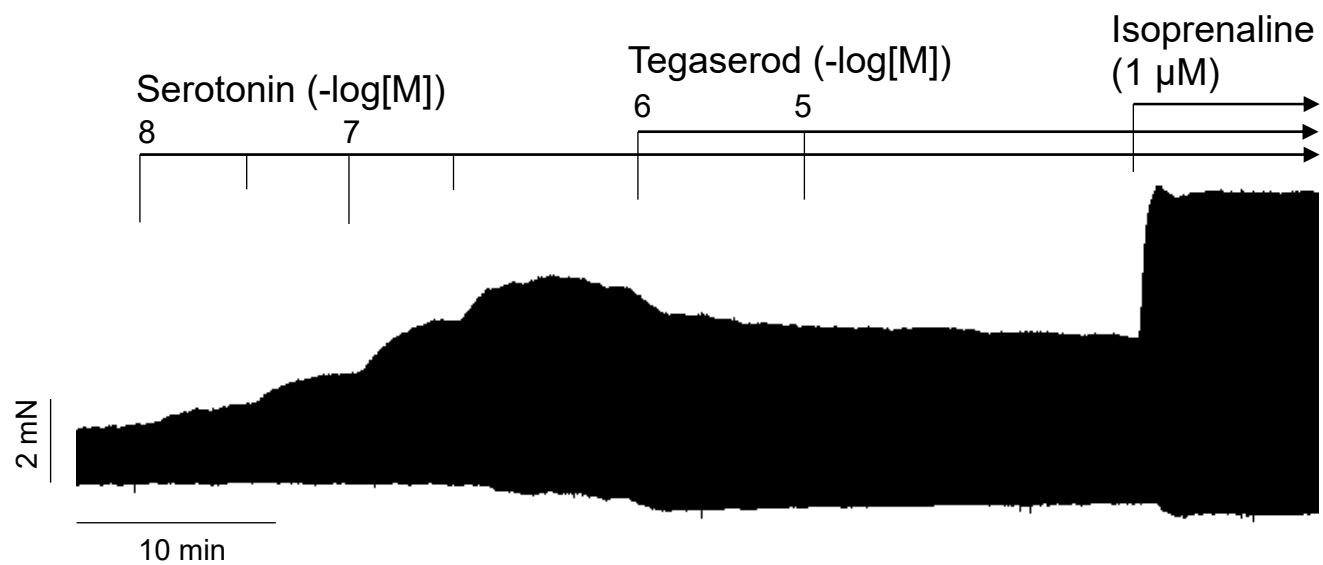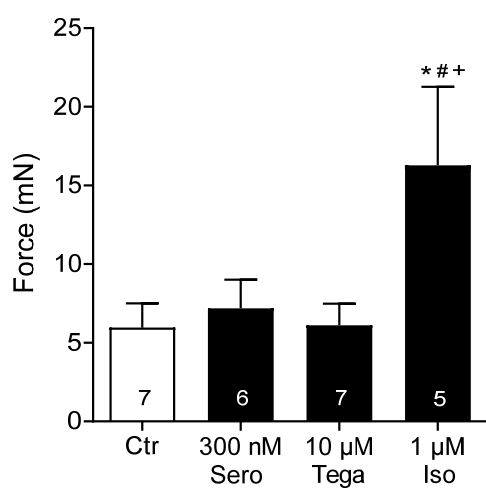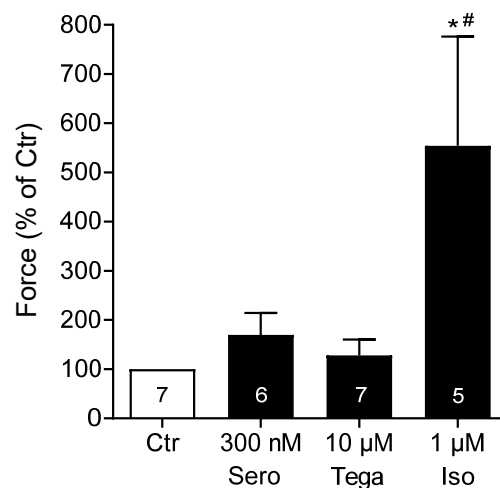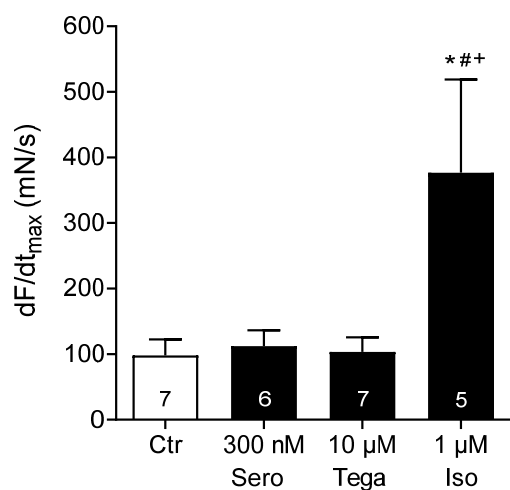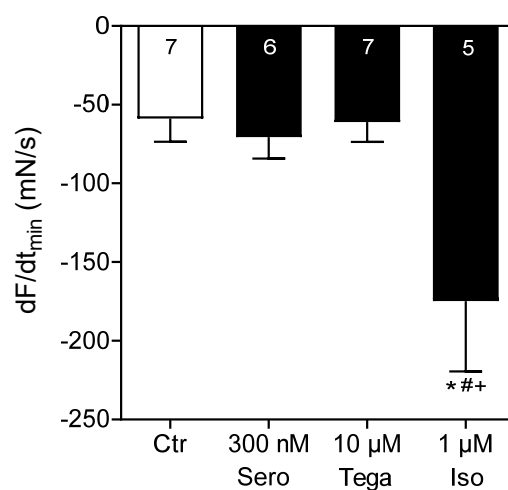

Supplement: Supplementary file 1 [file ijms-25-11133-s001.zip › ijms-3227703-supplementary.pdf]
